# A Case of Concurrent Autoimmune Thyroiditis and Adrenal Insufficiency Following Nivolumab Therapy for Advanced Renal Cell Carcinoma

**DOI:** 10.7759/cureus.109548

**Published:** 2026-05-24

**Authors:** Gillian D Dooley, Henry Li, Ihunanya Agomuoh, Fahd Al-Saghir, Camelia Arsene

**Affiliations:** 1 Medical School, Ross University School of Medicine, Pontiac, USA; 2 Medical School, Wayne State University School of Medicine, Pontiac, USA; 3 Nephrology, Michigan Kidney Consultants, P.C., Pontiac, USA; 4 Internal Medicine, St. Joseph Mercy Oakland Hospital, Pontiac, USA

**Keywords:** adrenal insufficiency, hypophysitis, immune checkpoint inhibitor, immune-related adverse events, immuno-chemotherapy, nephrology, nivolumab, onco-nephrology, renal cell carcinoma, thyroiditis

## Abstract

Nivolumab, a programmed death-1 (PD-1) immune checkpoint inhibitor (ICI), improves outcomes in advanced renal cell carcinoma (RCC) by enhancing antitumor T-cell activity. Immune activation may result in immune-related adverse events, including endocrine dysfunction. Thyroid abnormalities are commonly observed, whereas adrenal insufficiency is less frequent but clinically significant. We report a 59-year-old man with metastatic chromophobe RCC who developed autoimmune thyroiditis followed by secondary adrenal insufficiency after treatment with nivolumab. Thyroid dysfunction occurred approximately one month after therapy initiation, and adrenal insufficiency developed eight months later, presenting with orthostatic hypotension and severe hyponatremia. Laboratory evaluation demonstrated undetectable cortisol with inappropriately normal adrenocorticotropic hormone, consistent with ICI-related hypophysitis. The hyponatremia improved after fludrocortisone was added to glucocorticoid therapy due to clinical refractoriness. This case highlights the rare occurrence of concurrent endocrine immune-related adverse events during PD-1 inhibitor therapy and underscores the importance of vigilant longitudinal monitoring of endocrine function in patients receiving ICI. Early recognition and prompt multidisciplinary management are essential to prevent life-threatening complications while allowing continuation of effective oncologic therapy.

## Introduction

Immune checkpoint inhibitors (ICIs) targeting programmed cell death protein-1 (PD-1), including nivolumab, have become a standard therapy option for advanced renal cell carcinoma (RCC) [1]. Nivolumab is a fully human immunoglobulin G4 monoclonal antibody that enhances T-cell-mediated antitumor immunity by blocking PD-1 interaction with programmed death ligand-1 (PD-L1) and PD-L2 [1,2]. Its clinical efficacy was established in the landmark CheckMate 025 randomized phase 3 trial, which demonstrated significantly improved overall survival with nivolumab compared with everolimus in patients with previously treated advanced RCC, leading to its widespread adoption in clinical practice [3]. 

The European Society for Medical Oncology guidelines recommend using nivolumab mainly as part of combination therapy for patients with advanced or metastatic clear-cell RCC. Common first-line options include nivolumab with ipilimumab for patients with intermediate or poor risk, and nivolumab with cabozantinib for patients across all risk categories. These immunotherapy-based combinations have been shown to improve both overall survival and progression-free survival compared to sunitinib [4].

While nivolumab provides a meaningful survival benefit, its mechanism of immune activation predisposes patients to immune-related adverse events (irAEs), with the endocrine system among the most frequently affected [5-7]. Thyroid dysfunction is commonly reported after initiation of a PD-1 inhibitor at a rate of 9.9%, while adrenal insufficiency and hypophysitis remain less frequent at a rate of 4.7% but are potentially life-threatening if unrecognized [8-13]. However, these irAEs are not mutually exclusive, and concurrent endocrinopathies can occur at a rate of 1.4% [13]. While the current guidelines recommend routine endocrine surveillance during therapy, the presence of one irAE is not an indication to cease monitoring for others [13]. Here, we present a rare case of autoimmune thyroiditis followed by secondary adrenal insufficiency after nivolumab therapy for RCC to emphasize the need for constant endocrine monitoring during the use of PD 1 inhibitors.

## Case presentation

A 59-year-old man with a history of non-clear cell RCC diagnosed in 2022 underwent right radical nephrectomy. An inferior vena cava (IVC) tumor thrombus was noted and persisted postoperatively. In February 2024, surveillance computed tomography (CT) imaging revealed significant retroperitoneal lymphadenopathy and a new 1.5 cm hepatic lesion, which biopsy confirmed as metastatic chromophobe RCC. Cabozantinib (60 mg daily) was initiated on May 11, 2024, and nivolumab infusions began on October 15, 2024. 

**Figure 1:**
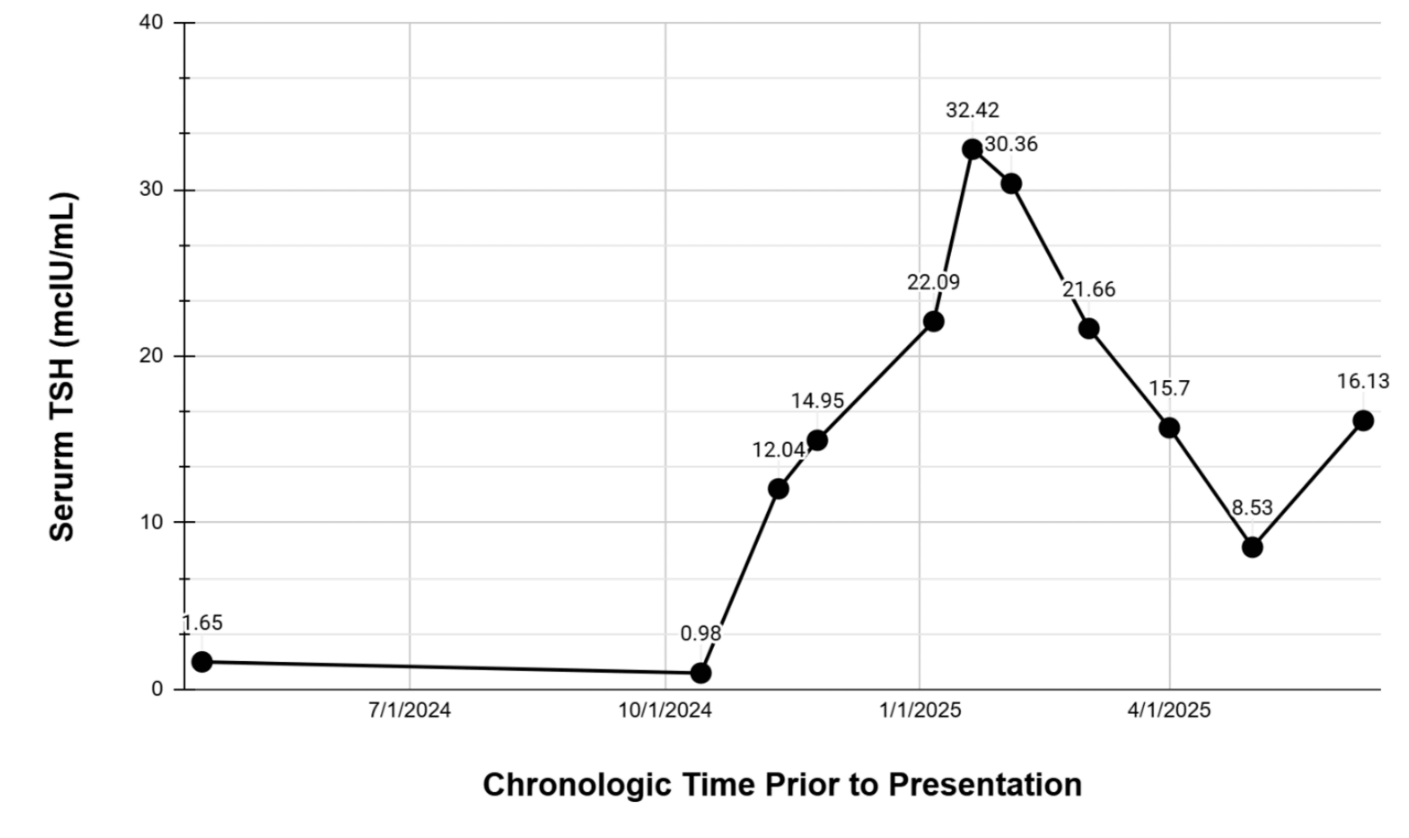
Serum thyroid-stimulating hormone (TSH) levels over time

One month following nivolumab initiation in November 2024, progressive hypothyroidism developed with rising thyroid-stimulating hormone (TSH) levels over several months. Levothyroxine therapy led to partial biochemical improvement. Longitudinal thyroid function values are shown in Figure 1.

**Figure 2:**
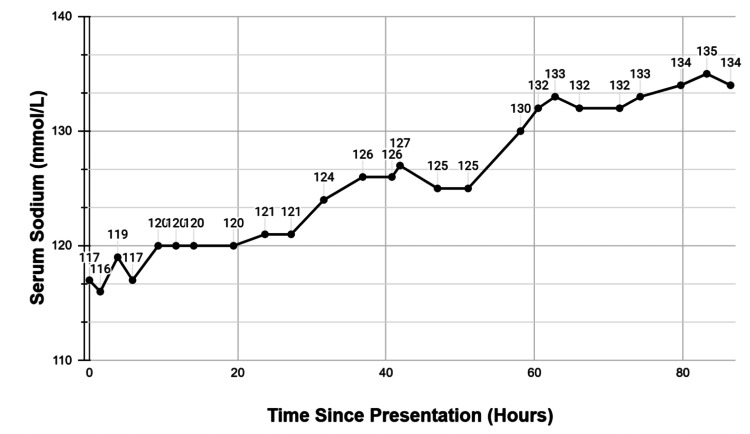
Serum sodium levels

On June 10, 2025, the patient presented with fatigue, nausea, vomiting, diarrhea, and severe orthostatic hypotension. Laboratory testing revealed acute hyponatremia (serum sodium 117 millimoles per liter (mmol/L)), requiring intensive care admission and hypertonic saline infusion. Sodium trends are shown in Figure 2. 

**Figure 3:**
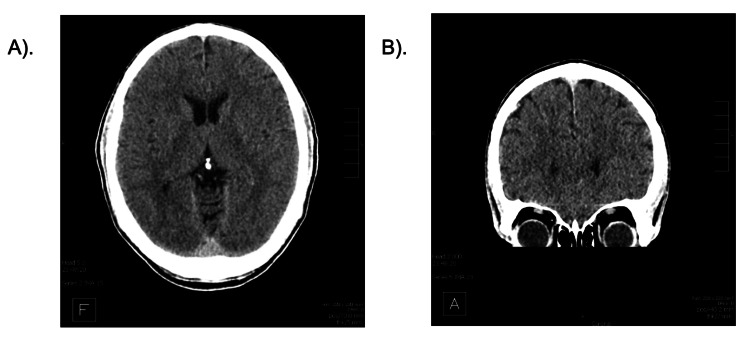
Computed tomography of the head without contrast in transverse plane (A) and coronal plane (B).

CT of the head without contrast (June 10, 2025) demonstrated no acute intracranial abnormality (Figure 3).

**Figure 4:**
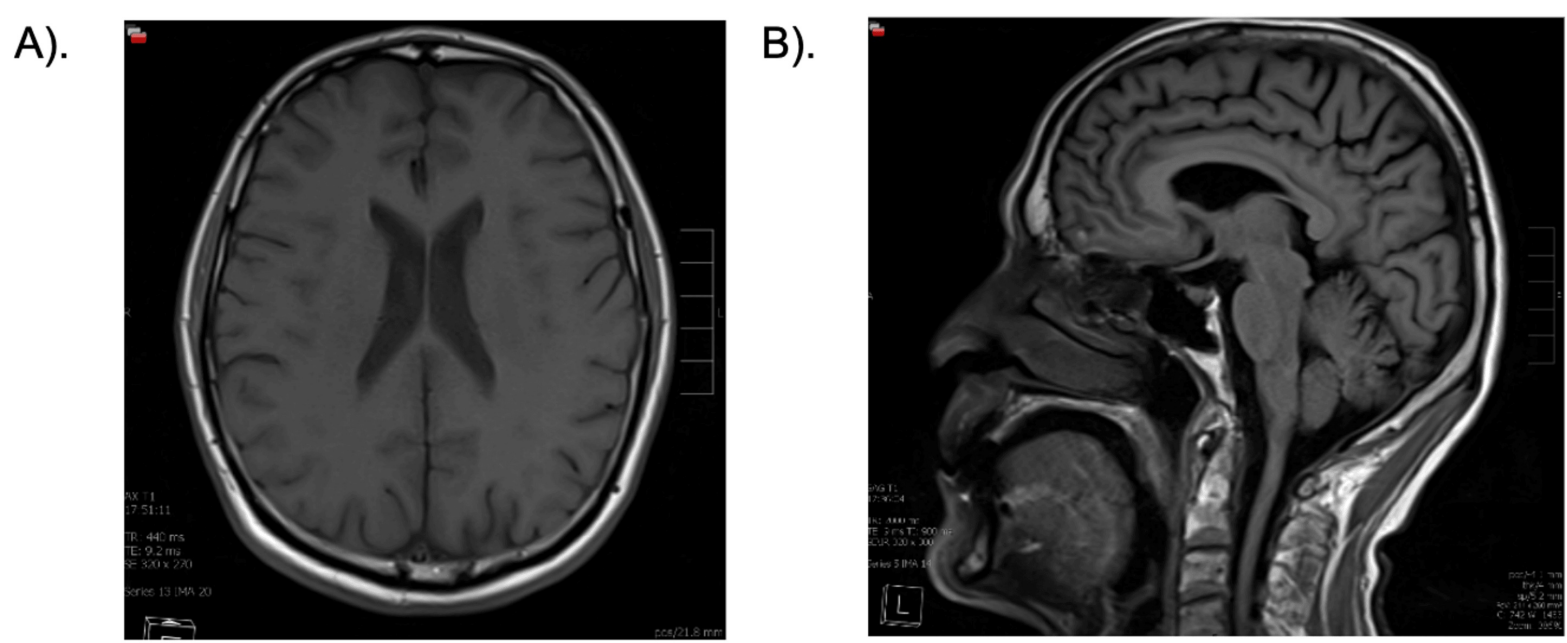
Magnetic resonance imaging of the brain with and without contrast in the transverse (A) and sagittal views (B).

Magnetic resonance imaging (MRI) of the brain with and without contrast (Figure 4) was ordered on June 12, 2025, to rule out malignant tumor spread and pituitary apoplexy. 

The MRI showed no evidence of metastatic spread since all sulci, ventricles, and cisterns were normal in size and configuration, there was no mass effect, midline shift, hydrocephalus, or abnormal extra-axial collection. In addition, the pituitary gland was not enlarged and had no abnormal intensities.

Pituitary apoplexy was similarly ruled out due to the lack of evidence of hemorrhage or infarct within the pituitary gland on the MRI. The patient lacked any symptoms, such as severe headache or vision changes.

Endocrine testing demonstrated serum cortisol <0.4 µg/dL (June 10, 2025) and adrenocorticotropic hormone (ACTH) 12 pg/mL (June 11, 2025), consistent with secondary adrenal insufficiency, likely due to ICI-related hypophysitis (Table 1). Steroid-induced adrenal insufficiency was ruled out due to the lack of long-term steroid use. The patient denied any use of glucocorticoids; however, the patient had recent chemotherapy with hydrocortisone. Chart review found no evidence of other glucocorticoid use, such as dexamethasone.

**Table 1:** Laboratory findings Note: Reference ranges are laboratory standard adult values.

Test name	Value	Biological reference range
Free thyroxine (Free T4)	0.64–0.85	0.8–1.8 ng/dL
Thyroid-stimulating hormone (TSH)	0.98–32.42	0.4–4.5 mIU/L
Sodium	116–145	135–145 mmol/L
Cortisol (AM)	<0.4	5–25 µg/dL
Adrenocorticotropic hormone	12	7–63 pg/mL
Aldosterone	9.7	4–31 ng/dL
Testosterone	109	300–1000 ng/dL
Luteinizing hormone	36.9	1.7–8.6 mIU/mL
Follicle-stimulating hormone	25.3	1.5–12.4 mIU/mL
Direct renin	<2.1	3.1-57.1pg/mL

Syndrome of inappropriate antidiuretic hormone (SIADH) secretion was considered as a possible differential diagnosis given that at the time of the severe hyponatremia of 117 mmol/L, the serum osmolality was 249 mOsm/kg H₂O, the urine osmolality was 348 mOsm/kg H₂O, and the urine sodium was 80 mmol/L. However, SIADH traditionally presents as euvolemic and does not result in orthostatic hypertension. In addition, patients with low ACTH and low cortisol make the diagnosis of hypophysitis more likely.

The patient was treated with 1L of normal saline, 3% hypertonic saline infusion, and hydrocortisone (50 mg AM, 10 mg PM) to correct his sodium levels and low cortisol levels (<0.04µg/dL), but hyponatremia persisted. Secondary adrenal insufficiency typically results in isolated cortisol deficiency; however, in more complex or refractory cases, fludrocortisone can be considered to address ongoing symptoms, such as postural dizziness, salt craving, or hypotension. In these situations, it acts with mineralocorticoid-like effects, helping to support sodium retention and maintain fluid balance [14]. The patient was started on 0.1 mg Fludrocortisone daily, leading to an increase in sodium from 117 mmol/L to 134 mmol/L by discharge. Orthostatic symptoms resolved. He was discharged on oral hydrocortisone (50 mg AM, 10 mg PM) and fludrocortisone 0.1 mg daily. 

The use of fludrocortisone is very atypical. The reasoning behind the administration of the fludrocortisone was due to refractory severe hyponatremia that was not responsive to 1 L of normal saline, 3% hypertonic saline infusion, 249 mmol/L serum osmolality, 348 mmol/L urine osmolality, and 80 mmol/L urine sodium.

His cortisol level was <0.04 µg/dL, and he was started on IV hydrocortisone 50 mg every 12 hours. His plasma renin and aldosterone came back low. Once the fludrocortisone was added, his orthostasis improved, and his sodium increased to 134 mmol/L at the time of discharge.

The patient followed up with nephrology outpatient post-admission, and orthostatic symptoms and hyponatremia further resolved. It was recommended that he continue hydrocortisone and fludrocortisone daily and be monitored for electrolytes and renal function. 

The patient will likely be receiving steroid treatment and thyroid hormone for the foreseeable future, as he is currently, especially since he is still compliant with his ICI treatment. The patient is asymptomatic with his daily 15 mg dose of hydrocortisone and 75 mcg of levothyroxine. 

## Discussion

PD-1 functions as a key ICI that limits autoreactive T-cell activation and maintains peripheral immune tolerance [1,2]. Initially identified in 1992 at Kyoto University, subsequent experimental studies demonstrated that mice lacking the PD-1 gene developed spontaneous autoimmune disease, highlighting its essential role in preventing immune responses against self-antigens [1,2]. The PD-1 receptor is expressed primarily on activated T lymphocytes and exerts its inhibitory effects when engaged by PD-L1 or PD-L2. This interaction suppresses intracellular signaling through recruitment of phosphatases and modulation of downstream signaling pathways, ultimately reducing T-cell proliferation and cytokine production [1,2]. Therapeutic blockade of this pathway enhances antitumor immunity but simultaneously disrupts immune self-tolerance, predisposing patients to immune-related adverse events, particularly endocrinopathies. Because the same PDL1 is expressed in many tissues throughout the body, such as those within the thyroid and pituitary endocrine tissue. The suppression of these immune checkpoint signal modulators results in increased activation of the adaptive immune system in non-malignant tissue. Therefore, by downregulating the normal protective measures to prevent autoimmune activation, PD-1 inhibitors increase the likelihood of the activation of T cells on one's own tissue [15].

Endocrine immune-related adverse events are among the most frequently reported toxicities associated with ICI, with thyroid dysfunction representing the most common manifestation [5-8]. ICI-associated thyroiditis often follows a biphasic course characterized by transient thyrotoxicosis followed by persistent hypothyroidism, although patients may present directly with biochemical hypothyroidism as seen in this case [5,6]. Our patient developed biochemical hypothyroidism approximately one month after initiation of nivolumab, consistent with the typical early onset pattern described in prior studies and reflecting immune-mediated destruction of thyroid tissue.

Secondary adrenal insufficiency typically presents with acute or sub-acute symptoms (median time to onset three months, range 1.5 weeks-30 months) secondary to pituitary inflammation and/or associated hormone loss. The patient in the case presented eight months after initiation of therapy and manifested with severe hyponatremia, orthostatic hypotension, and systemic symptoms requiring intensive care monitoring. Laboratory findings of undetectable cortisol with inappropriately normal ACTH supported a diagnosis of ICI-related hypophysitis. Unlike primary adrenal insufficiency, hypophysitis associated with PD-1 inhibitors frequently presents with isolated ACTH deficiency, while other pituitary axes may remain partially preserved [10,16]. Prior observational data suggest that hypophysitis associated with PD-1 inhibitors tends to occur later than that seen with cytotoxic T-lymphocyte antigen-4 blockade and may demonstrate variable clinical severity and delayed recognition due to nonspecific symptoms [10,11,16].

Previous studies of PD-1 inhibitor-associated hypophysitis further demonstrate that isolated ACTH deficiency is the predominant presentation and that pituitary MRI may remain normal despite clinically significant endocrine dysfunction, which parallels the findings observed in this patient [17,18]. In traditionally lymphocytic hypophysitis, the most common form of primary hypophysitis, representing 76-86% of cases, typical MRI findings included pituitary contrast enhancement (63%), symmetric pituitary enlargement (60%), pituitary stalk thickening (58%), a sellar mass or suprasellar extension (58%), and loss of the posterior pituitary hyperintensity (50%) [17,18]. However, for immune-related adverse events secondary to immune checkpoint inhibitors, recent findings suggest that the MRI is normal for most cases of isolated adrenocorticotropic hormone deficiency and may be normal in half of the patients with immune-related hypophysitis. Thus, the lack of signs of pathologic processes on the MRI does not rule out irAE [17].

Unlike many previously reported cases describing simultaneous endocrine toxicities, this patient demonstrated a sequential immune-related adverse event pattern, with thyroid dysfunction preceding pituitary-adrenal axis failure. This temporal dissociation reinforces the concept that endocrine immune-related toxicities may evolve independently over prolonged treatment courses and should not be considered static once an initial endocrinopathy is diagnosed. Emerging evidence suggests that patient-specific immune susceptibility and tumor-related immune activation may influence the timing and pattern of endocrine injury, although the mechanisms underlying delayed endocrine toxicity remain incompletely understood [16].

Although mineralocorticoid replacement is generally unnecessary in secondary adrenal insufficiency due to preserved renin-angiotensin-aldosterone axis function, the persistent hyponatremia despite glucocorticoid replacement improved only after initiation of fludrocortisone. This phenomenon may be explained by the difference in potency for mineralocorticoid receptors. While hydrocortisone has both mineralocorticoid and glucocorticoid properties, fludrocortisone has much greater potency as a mineralocorticoid compared to hydrocortisone in a ratio of 125-150:1, respectively [19]. This response to fludrocortisone over hydrocortisone further highlights the potential heterogeneity of hypothalamic-pituitary-adrenal axis dysfunction following immune-mediated injury. Similar atypical clinical responses have been described in cases of ICI-associated endocrine toxicity and may reflect overlapping mechanisms of cytokine-mediated renal salt handling, volume depletion, or partial adrenal dysfunction [10,16]. 

While mineralocorticoid deficiency is classically associated with primary adrenal insufficiency, the clinical response observed in this patient suggests that electrolyte abnormalities during ICI-related hypophysitis may not always conform to traditional physiologic expectations. Severe hyponatremia in this setting is likely multifactorial, including glucocorticoid deficiency-mediated increases in antidiuretic hormone (ADH) secretion, reduced effective circulating volume, and impaired free water clearance. Similar atypical electrolyte disturbances and variable responses to hormone replacement have been described in ICI-related endocrinopathies, supporting an individualized treatment approach based on clinical response rather than strict adherence to conventional primary versus secondary adrenal insufficiency paradigms [10,16].

Finally, this case expands the reported clinical spectrum of endocrine immune-related toxicity in metastatic chromophobe RCC, a population less frequently represented in ICI literature compared with melanoma [8]. The literature review emphasizes that endocrine immune-related adverse events may occur months after therapy initiation and can present with nonspecific symptoms, highlighting the importance of continued endocrine surveillance throughout treatment rather than symptom-driven testing alone. The current literature recommends preventing endocrine irAEs, screening for thyroid disorders, and hypophysitis due to their high incidence rates. This includes initial fasting levels of cortisol, TSH, and free T4 and T3. TSH, fT3, and fT4 should be measured at every infusion during the first three months and every three to six months thereafter [20]. The availability of serial laboratory measurements preceding clinical decompensation further underscores the importance of proactive endocrine surveillance during immunotherapy. Coordination within subspecialty teams assisted in the formation of an appropriate care plan. A list of possible diagnoses, including malignant tumor spread, syndrome of inappropriate ADH secretion, pituitary apoplexy, and steroid-induced adrenal insufficiency, was developed and narrowed with input from the oncology, endocrinology, nephrology, and primary care teams. This team-based approach remains essential for early detection and successful management of endocrine immune-related adverse events while allowing continuation of effective anticancer therapy.

## Conclusions

This case describes a subacute presentation of autoimmune thyroiditis and secondary adrenal insufficiency following nivolumab therapy for metastatic RCC. The clinical course demonstrates that endocrine immune-related adverse events may occur months apart and present with severe metabolic complications. However, the degree of temporal separation as seen in our case supports the idea that endocrine immune-related toxicities can develop independently over extended treatment periods and should not be viewed as finite after an initial endocrinopathy is identified. Continuous vigilance is required to prevent the sequelae of additional irAEs; persistent laboratory monitoring, timely endocrine evaluation, and individualized hormone replacement are essential to ensure patient safety during ICI therapy.
